# Are “Global Measures” of Psychological Intimate Partner Violence Against Women Really Comparable? A Measurement Invariance Analysis of Controlling Behaviors in 19 Low- and Middle- Income Countries

**DOI:** 10.21203/rs.3.rs-4963461/v1

**Published:** 2024-09-25

**Authors:** Kathryn M. Yount, Erin Johnson, Nadine Kaslow, Yuk Fai Cheong

**Affiliations:** Emory University; Emory University; Emory University; Emory University

**Keywords:** controlling behavior, measurement invariance, psychological intimate partner violence against women, low- and middle-income countries, sustainable development goal 5.2.1

## Abstract

**Background:**

One third of adult women report lifetime psychological intimate partner violence (IPV). Controlling behavior is a common dimension of psychological IPV; however, evidence is mixed on its cross-national and cross-time measurement invariance, limiting its use to monitor Sustainable Development Goal (SDG) 5.2.1, to eliminate all forms of violence against women. We explored easier-to-modify survey-design features and harder-to-modify individual-level and national-level characteristics that may account for non-invariance of these controlling-behavior items.

**Methods:**

We analyzed data on five controlling behaviors administered to 373,167 ever-partnered women 15–49 years in 19 low- or middle-income countries in which at least two national Demographic and Health Surveys were administered during 2005–2019. We performed multiple-group confirmatory factor analysis (MGCFA) to test for exact forms of invariance and alignment optimization (AO) to test for approximate invariance across 7–9 survey-design groups, defined by the number of preceding questionnaire modules (to proxy respondent burden) and weeks of interviewer training (to proxy interviewer skills). Adjustment for covariates in the MGCFA assessed whether individual- and national-level characteristics could account for any observed non-invariance across survey-design groups.

**Results:**

In MGCFA without covariates, configural invariance of the controlling-behavior items was observed across survey-design groups. Exact invariance, partial invariance (with 20% of parameter estimates freed), and approximate invariance were *not* observed across groups. In adjusted MGCFA, neither woman-level covariates (schooling, attitudes about IPV against women) nor national-level covariates (women’s mean schooling, mean attitudes about IPV against women, gender-related legal environment) alone or combined accounted for the non-invariance of controlling-behavior items across survey-design groups.

**Conclusions:**

Comparing estimates for controlling behavior across country, time, and survey design variations warrants caution. Standardizing questionnaire length and interviewer training may improve the invariance of these items. Other characteristics, like ethnicity and language, may account for the non-invariance of controlling-behavior items across survey-design groups and should be tested. Current controlling-behavior items should be refined to enhance their comparability, and new controlling-behavior items should be identified and tested to improve the item set’s content validity. Given current evidence of the high prevalence and health impacts of psychological IPV against women, advancing this research agenda is needed to monitor SDG 5.2.1.

## Background

An estimated 33% of women 16 years or older report experiencing lifetime psychological intimate partner violence (IPV), and women in community-based samples report the highest lifetime exposure, at 41% (1). Psychological IPV is a multi-dimensional construct that is variously defined (Appendix Table 1). Common dimensions include verbal aggression, coercive control and controlling behavior, and in some definitions, economic coercion. In low- and middle-income countries (LMICs), verbal aggression and economic coercion have received somewhat more scholarly attention (2–4). *Coercive control and controlling behavior*, however, lacks a standard definition and is measured using an array of scales (5). We propose the following working definition of controlling behavior: *one partner’s credible demands for compliance with behaviors that systematically constrain the other partner’s actions, relationships, and activities*.

In LMICs, the Demographic and Health Surveys (DHS) have included 5–6 questions (or items) on controlling behavior, including: jealousy or anger for *talking to other men*; accusations of *infidelity*; not permitting the respondent *to meet female friends*; trying to limit the respondent’s *contact with family*; insisting on knowing where the respondent is at all times; and in earlier rounds, not trusting the respondent with money (emphasis added). Vis-à-vis our working definition, these items focus on behavior that constrains a female partner’s *relationships*. During 2000–2021, 110 DHS in 57 LMICs spanning Asia, Africa, Eastern Europe, Latin America and the Caribbean, the Middle East, and Oceania have produced national-level data on a partner’s use of controlling behavior with women (6), offering the most geographically comprehensive data with responses to a common set of items for this dimension of psychological IPV.

One question is whether these controlling-behavior items are invariant cross-nationally and across time, meaning they can be used validly to compare mean estimates of controlling behavior across countries and calendar years. This question is relevant, given the call to monitor Sustainable Development Goal Target 5.2, to eliminate all forms of violence against all women and girls, using indicator 5.2.1, the proportion of ever-partnered women 15 years and older subjected to physical, sexual or psychological violence by an intimate partner in the prior 12 months, by form of violence and by age. To address this question, we undertook analyses to assess the recent cross-national invariance, and separately, the cross-national and cross-time invariance of the five DHS controlling-behavior items, and the findings across these analyses were mixed. In the first analysis, these items were administered to 136,693 ever-partnered women of reproductive age who took part in 36 DHS in 36 LMICs during 2012–2018; the controlling-behavior items lacked exact *cross-national invariance* but achieved *approximate cross-national invariance* (7). In the second analysis, these controlling-behaviors items were administered to 373,167 women who took part in one of 42 DHS conducted 1–12 years apart in 19 LMICs during 2005–2019; these items achieved *exact cross-time invariance within* 11 countries, but neither *exact* nor *approximate cross-country, cross-time invariance* in the pooled analysis of these 11 countries (8).

The present analysis aims to examine why the DHS controlling-behavior item set may lack cross-country, cross-time invariance. First, we tested whether cross-country, cross-time invariance of the item set was observed across sample groups that differed on two survey-design features considered important for the quality of self-reported data on experiences of IPV: number of preceding questionnaire modules as a proxy for respondent burden, and number of weeks of interviewer training as a proxy for interviewer skills (9–11). If non-invariance of the items was observed across survey-design groups, we examined whether adjusting for variation in woman-level characteristics, population-level characteristics, and the national, gender-related legal environment—accounted for the non-invariance. Findings suggest the potential need for a multifaceted strategy involving 1) consistent questionnaire length and interviewer training; 2) an analytical strategy for post-hoc adjustment of existing controlling-behavior data; and more importantly 3) refinements of the DHS controlling-behavior items to improve their content validity vis-à-vis the above working definition and valid cross-national use to monitor SDG 5.2, to eliminate all violence against women and girls.

## Methods

### Study Setting and Sample

The general study setting was low- and middle-income countries in which a domestic violence module was administered as part of the Demographic and Health Surveys (DHS). The DHS, a program supported by the U.S. Agency for International Development (USAID) and operating in more than 90 LMICs, collects data on population and health, including IPV against ever-partnered women of reproductive age. A domestic violence module (DVM) is administered in a sub-sample of between 15% and 100% of households that are interviewed in the parent DHS. In selected households, one woman 15–49 years is randomly selected per household and interviewed. We restricted the sample of DHS to those that had administered DVM versions V through VII during the period 2005–2019. This restriction ensured similarity in the number and wording of IPV items across administrations of the DHS both within and across included LMICs. Within this frame, final samples included ever-partnered women 15–49 years from 18 LMICs and ever-partnered women 18–49 years from one LMIC, or 19 LMICs total in which at least two DHS were conducted 1 to 12 years apart during 2005–2019 and included the same five controlling-behavior items.

The total sample of eligible women for this analysis included 380,012 DHS participants who were selected and administered the DVM and were not skipped out of the IPV items due to reporting a never-partnered status. Of these participants, 6,845 were missing data on all controlling-behaviors items, bringing the final sample for analysis to 373,167 ever-partnered women 15–49 years across 19 countries and 42 DHS. All DHS samples were downloaded with written permission from the DHS program.

### Controlling Behavior Items

Questions on controlling behavior typically appear near the beginning of the DVM, which typically is the final or near final module in the DHS woman questionnaire. To measure controlling behaviors, participants were asked whether their husband or partner did or did not do each of five behaviors without reference to a time frame: is jealous if the respondent talked to other men, frequently accuses the respondent of being unfaithful, does not permit the respondent to meet her female friends, tries to limit the respondent’s contact with her family, and insists on knowing where the respondent is at all times. Response options were “yes” or “no” for experiencing each behavior by the husband or partner.

### Survey Design Groups: More Modifiable Potential Sources of Non-Invariance in Controlling Behavior Items

To create the survey-design groups for invariance testing of the controlling-behavior items, we combined information on the number of questionnaire modules preceding the DVM as a proxy for respondent burden and the number of weeks of interviewer training as a proxy for interviewer skill. We, then, categorized each DHS dataset into one of nine survey-design groups, defined by creating tertiles for each of the two survey-design variables. Survey-design groups ranged from the *most favorable*, defined as having the *fewest* preceding questionnaire modules (nine) and the *most* interviewer training (six weeks) to the *least favorable*, defined as having the *most* preceding questionnaire modules [15] and the *least* interviewer training (one week). Given assertions of the importance of minimizing respondent burden and of maximizing interview skill for the quality of reported data on IPV (9, 12), we expected that the controlling-behavior items would not be exactly invariant across these survey-design groups.

#### Less-easily-modified Individual and National Characteristics that may Reduce Any Observed Non-Invariance of Controlling Behavior Items Across Survey-Design Groups

To adjust for factors that may reduce any observed non-invariance across the survey-design groups (13, 14), we considered woman-level, population-level, and national-level covariates that we hypothesized may be associated with differences in respondents’ interpretation of or willingness to report exposure to controlling behaviors (15, 16). Woman-level covariates included her completed years of schooling and whether (= 1) or not (= 0) she justified IPV against women for any of five situations: wife burns the food, wife argues with her partner, wife goes out without telling her partner, wife neglects the children, and wife refuses to have sex with her partner. The attitudinal variable was computed as an average across the five dichotomous items, producing a score between 0 and 1 for each respondent, with higher scores indicating higher justification of IPV against women (17, 18). National-level covariates included women’s mean completed years of schooling across all respondents for each country and DHS, women’s mean IPV against women attitudes across all respondents for each country and DHS, and the World Bank’s Women, Business, and Law Index (WBLI), which scores 35 data points across eight indicators of four or five binary questions on laws and regulations affecting women’s economic opportunity, with each indicator capturing a different phase of a woman’s career (19). The eight indicators cover gender equality and protections related to: mobility, the workplace, pay and job access, marriage, parental leave, entrepreneurship, assets ownership and management, and pensions. The composite WBLI scores represent unweighted means of each indicator, scaled to 100, with 100 representing the highest possible gender-equality score.

### Statistical Analysis

**Figure 1.**
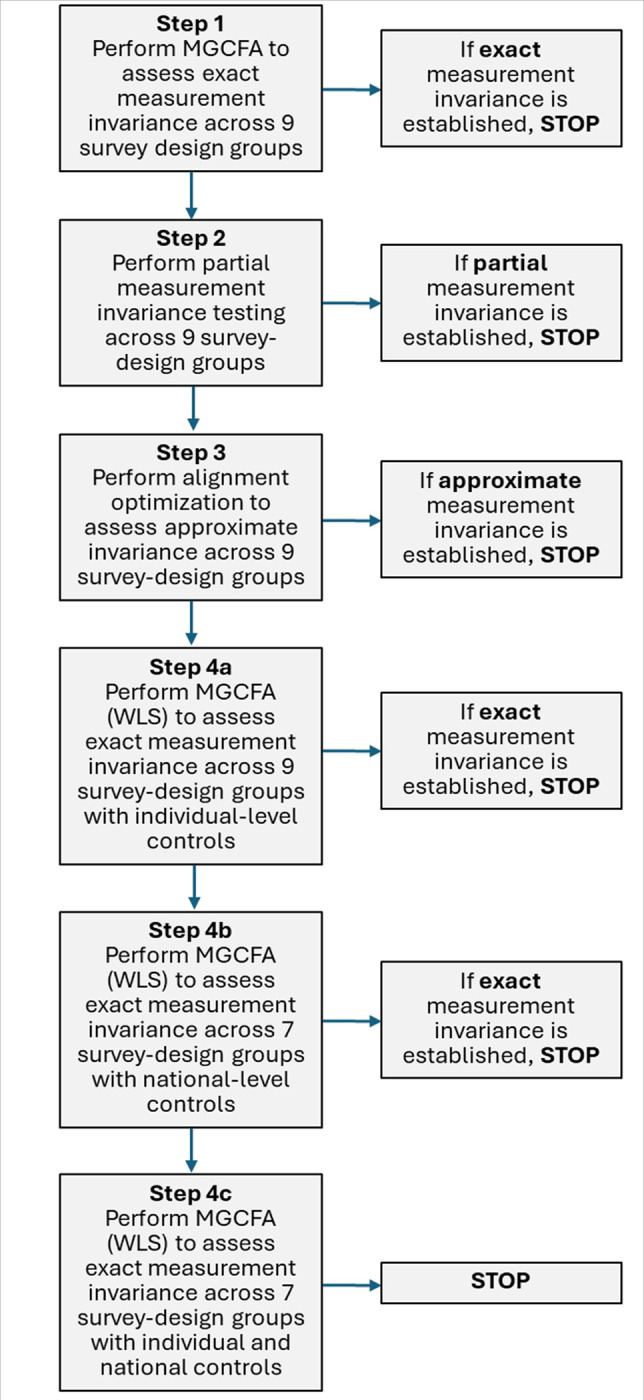
A priori tests and decision-rules for the analysis

We used STATA 17 for data cleaning and management (20) and MPlus 8 for measurement invariance testing (21, 22). After constructing variables and performing descriptive analysis, we followed a series of predefined steps in this analysis (Fig. 1). In Step 1, we tested the measurement invariance of the five controlling-behavior items across the nine survey-design groups. We performed multiple group confirmatory factor analysis (MGCFA), using weighted least squares (WLS) estimation and DHS-generated probability weights and cluster variables in all models to account for the probabilities of selection into the sample and for the clustering of responses in primary sampling units (PSUs) and PSU segments. We assessed the fit of the configural model, in which all loadings and thresholds were estimated freely across the nine survey-design groups, using several indices: chi-square (χ^2^), Root Mean Square Error of Approximation (RMSEA, adequate fit ≤ 0.08, good fit ≤ 0.05), and the Comparative Fit Index (CFI ≥ 0.95) and Tucker-Lewis Index (TLI ≥ 0.95) (23). We then compared the fit of the configural model with that of the nested scalar model, in which loadings and thresholds were constrained to be equal across the nine survey-design groups. To do so, we computed the chi-square difference test (Δχ^2^) and changes in the CFI (ΔCFI) and RMSEA (ΔRMSEA), using the general guidelines of Cheung and Rensvold (24) and Chen (25) and cut-off values proposed by Meade et al. (26) and Martín-Fernández et al. (27), which currently are the most conservative for assessing changes in fit indices (unconstrained model – constrained model): ΔCFI ≤ .002 and ΔRMSEA ≤ .007. The performance of these cut-offs with categorical data tends to be similar to maximum likelihood-based procedures when the sample size is large, and the items are not normally distributed.

If evidence was lacking for scalar invariance of the controlling-behavior item set, we tested for the partial invariance of the item set (Step 2, Fig. 1). To do so, we examined modification indices to identify model parameters that were non-invariant across survey-design groups (28). Large modification indices helped us to identify model parameters that may be contributing substantially to non-invariance. We then freed between-group equality constraints of factor loadings and thresholds together as they had to be tested simultaneously for binary indicators (28), starting with the parameters that had the largest modification indices. We then released the next set of between-group equality constraints and assessed the extent of improvement with the significance of the Χ^2^ difference test. We continued in this fashion until partial invariance was achieved or equality constraints had been released for 20% of parameters in the model (29, 30).

If the controlling behavior items showed a lack of scalar invariance across survey-design groups in Step 1, and a lack of partial invariance in Step 2, we proceeded to Step 3 (Fig. 1). In Step 3, we performed alignment optimization (AO) to test for the approximate invariance of this item set across survey-design groups (31, 32). AO relaxes the strict-invariance assumptions of MGCFA by allowing estimated group-specific model parameters to vary randomly from the estimated model parameters in the pooled dataset following a normal distribution. Each item-specific R^2^ value gauges the degree of invariance for each controlling-behavior item by indexing the proportion of variability in that item that the groups’ factor means and variances can account for. A higher R^2^ value for a controlling-behavior item indicates a higher level of item-specific invariance across groups. For the overall AO model, the criterion for approximate invariance was evidence that ≤ 25% of model parameters (loadings and thresholds) were non-invariant (31, 32).

If Step 3 did not provide evidence for approximate invariance of the controlling-behavior items, we proceeded to Step 4 (Fig. 1). In this step, we repeated the MGCFA from Step 1, this time controlling separately (Steps 4a and 4b) and then jointly (Step 4c) for woman-level and national-level covariates that may reduce the observed non-invariance across survey-design groups (10). Covariates of interest included woman-level variables for schooling attainment and attitudes about IPV against women, as well as national-level variables for women’s mean schooling attainment, women’s mean attitudes regarding IPV against women, and a composite score for the national legal environment, as measured by the Women, Business, and Law Index (19). In analyses with national-level covariates, two of the nine survey-design groups included one dataset each, lacking sufficient variability in the covariate for this analysis. For MGCFA with national-level covariates, we combined these two groups with adjacent groups, creating seven survey-design groups.

## Results

### Descriptive Characteristics of Included Datasets

Table 1 provides a description of the datasets and women included in this analysis, across survey-design groups. The number of DHS datasets per survey design group ranged from 1 to 10. Sample sizes of ever-partnered women of reproductive age ranged from 3,120 to 84,357 per survey design group. The number of weeks of enumerator (interviewer) training varied considerably across survey-design groups, ranging from a minimum of 1–3 weeks to a maximum of 5–6 weeks of training. The number of preceding questionnaire modules also ranged widely across survey-design groups, from 9–11 preceding modules to 14–15 preceding modules, reflecting large potential differences in respondent burden related to questionnaire length. The extent of justifying wife beating among sample women was low to moderate, with mean scores ranging from 0.07 to 0.51 across survey-design groups. The mean completed years of schooling for women also ranged widely, from 1.48 completed years to 11.21 completed years across survey design groups. Finally, the mean Women, Business, and Law Index Score ranged from a low of 31.9 to a high of 72.9 across survey design groups, suggesting substantial differences in the gender-related legal environment across countries represented in the survey-design groups.

### Step 1. MGCFA to Test for Configural and Scalar Invariance of Controlling-Behavior Items

Models 1a and 1b of Table 2 present findings for the MGCFA, in which configural and scalar invariance of the controlling-behavior items were tested across the nine survey-design groups in the absence of covariates. Fit statistics for the configural model were adequate to good (RMSEA = 0.056; CFI = 0.972; TLI = 0.944), suggesting that the controlling-behavior items loaded on a single factor across all survey-design groups (Table 2). To test for the scalar invariance of the controlling-behavior items, the ΔCFI between the configural model and the scalar model was not within its recommended range of ≤ 0.002 (26), and the χ^2^ difference test comparing the nested scalar model with the configural model was highly significant, at p < .0001. These results suggested that at least some of the estimated thresholds and/or loadings were not the same across survey-design groups.

### Step 2. Partial Invariance Testing

Since scalar invariance of the controlling-behavior item set was not observed in the MGCFA without covariates, we proceeded to MGCFA with partial invariance testing (Step 2) to assess whether a subset of controlling behavior items was found to be invariant across survey-design groups (results available upon request). After freeing 20% of model parameters, based on modification indices, the p-value for the chi-square difference test remained < 0.0001, indicating that evidence in favor of partial invariance could not be established.

### Step 3. Alignment Optimization to Test for Approximate Invariance of Controlling-Behavior Items

Table 3 presents the weighted average thresholds, weighted average loadings, and R^2^ statistics for each controlling behavior item from the AO analysis. Notably, the estimated intercepts and loadings for two controlling-behavior items—“does not permit the respondent to meet her female friends” and “tries to limit the respondent’s contact with her family”—displayed an extreme lack of invariance across survey-design groups, with R^2^ = 0 for both items. Moreover, R^2^ values were low for all controlling-behavior items, suggesting potential non-invariance of the item set more generally across survey-design groups.

To assess this possibility, Table 3 also presents the number and percentage of parameter estimates that were non-invariant in the AO analysis. Again, this figure provides a global assessment for the overall invariance of the controlling-behavior item set. The results showed that 12 of 45 (or 27% of) estimated loadings, 23 of 45 (or 51% of) estimated thresholds, and 35 of 90 (or 39% of) parameter estimates in total were non-invariant (Table 3). Using the recommended benchmark of 25% or fewer total non-invariant parameter estimates for trustworthy factor mean comparisons of controlling behavior across survey-design groups (31, 32), the results from AO suggested that the item set for controlling behaviors did not exhibit approximate measurement invariance across these groups.

### Step 4 (a-c). MGCFA with Adjustment for Individual- and National-Level Covariates

Table 2 also reports the MGCFA results across survey-design groups with the addition of covariates to the models. We entered, separately and then jointly, the woman-level covariates of schooling attainment and attitudes about wife beating, and national-level covariates for women’s mean schooling attainment, women’s mean attitudes about wife beating, and the Women, Business, and law Index score. As shown in Table 2, models 2 (a,b) through 9 (a,b), adding these covariates, either alone or jointly, did not account for the non-variance of the controlling-behavior items across survey-design groups. Although the ΔRMSEA improved and/or met the recommended threshold of ≤ 0.007 in six of the eight adjusted nested models (Table 2), the CFIs for the adjusted models were similar to or slightly worse than the CFIs for the models without covariates, and none of the ΔCFIs (CFI configural model – CFI scalar model) met the recommended threshold of ≤ 0.002 (26). Finally, significant χ^2^ difference tests between the configural and scalar models continued to be observed, regardless of the adjustment for covariates.

## Discussion

### Summary and Interpretation of Findings

This analysis is the first systematic assessment of potential reasons for measurement non-invariance of the five DHS controlling-behavior items administered during 2005–2019 in 19 LMICs and 42 DHS in which 373,167 ever-partnered women of reproductive age responded to at least one item (8). This analysis also is the first to use a carefully sequenced analytical strategy that tested, across seven to nine survey-design groups, for the following types of measurement invariance: first, configural and scalar invariance using MGCFA without covariates (Step 1); then, partial invariance using MGCFA and allowing the model parameters for some controlling-behavior items to vary across groups (Step 2); and then, approximate invariance of the full item set using alignment optimization (Step 3). In the absence of measurement invariance for the DHS controlling-behavior items across survey-design groups, we introduced in Step 4 a new technique to IPV research—MGCFA with covariate adjustment—to explore why measurement non-invariance of the item set was observed. This sequenced approach applied state-of-the-art guidance on the cross-cultural assessment of measurement invariance (33) to controlling-behavior items—a major dimension of psychological IPV against women that heightens the risk of other forms of IPV (34). This sequenced approach allowed us to understand more clearly the nature and sources of measurement non-invariance for this item set, providing guidance on how to improve the measurement of this dimension of psychological IPV against women, and more generally, on the cross-cultural measurement of psychological IPV against women.

In MGCFA in this large and diverse sample of women, this item set of five controlling behaviors achieved configural invariance across nine survey-design groups that varied in interviewer skill (weeks of training) and respondent burden (number of prior questionnaire modules). Importantly, this finding suggests that this DHS items appear to be related in the same direction to a single “controlling behavior” construct that has conceptual meaning across diverse survey-design environments (33).

Despite evidence for configural invariance, this controlling-behavior item set did *not* exhibit scalar invariance, or full score equivalence (33), across the nine survey-design groups. The achievement of scalar invariance would indicate that the DHS measure for controlling behaviors has the same loadings and thresholds across the nine survey-design groups and time period of 2005–2019. One interpretation of this finding is that the observed non-invariance of these items may be attributable to variability in respondent burden (questionnaire length) and interviewer skill (weeks of training).

Given the observational nature of this study, however, other explanations are possible. One alternative explanation is that the constraints for scalar invariance may be unrealistic in comparisons involving multiple groups, settings, cultures, and time periods (35). To address this issue, Byrne, Shavelson (36) introduced the concept of partial measurement invariance, in which a subset of parameters in MGCFA is constrained to be invariant, and another subset of parameters is allowed to vary across groups. When partial invariance is observed, the invariant subset of items can be compared across countries, cultures, groups, and/or time (36). However, limited guidance exists on the cutoff proportion of noninvariant parameters that can be released using this approach. In our assessment of the DHS controlling-behavior items, we were unable to establish partial invariance after releasing 20% of the parameter estimates.

In the absence of observing either scalar invariance or partial invariance of the controlling behavior items in MGCFA, we turned to alignment optimization (AO)—a novel approach to invariance testing in cross-cultural research on IPV. AO incorporates a simplicity function to discover the simplest model with the fewest noninvariant parameters and to estimate the factor mean and variance parameters in each group. In the application of AO here, two controlling-behavior items (“does not permit her to meet her female friends” and “tries to limit her contact with her family”) exhibited extreme non-invariance, as evidenced by R^2^ values of 0. This finding, alone, could suggest that item-specific non-equivalence contributed to non-invariance of the item set; however, the R^2^ values were relatively low (≤ 0.31) for all controlling behavior items in the set. In fact, 39% of all parameter estimates were found to be non-invariant. This percentage substantially exceeded the suggested threshold of 25% or fewer non-invariant parameters for trustworthy comparisons of factor means of controlling behavior across survey-design groups (29, 30). Thus, our findings from AO suggested that the DHS controlling-behavior item set was not approximately invariant across nine survey-design groups that captured survey conditions considered to be important for collecting high-quality data in IPV research (9).

A second explanation for the observed non-invariance in this controlling-behavior item set across survey-design groups is confounding; in other words, other covariates could explain the non-invariance across these groups (37). To explore this possibility, we estimated MGCFA with adjustment for theoretically relevant covariates at the woman level (completed years of schooling and attitudes about IPV against women) and the population or national level (e.g., national means for these variables and the gender-related legal environment). However, adjusting separately and jointly for these covariates did not reduce the observed non-invariance of the items across survey-design groups. Thus, despite adjustment for relevant individual and contextual factors, this item set remained measurement non-invariant across survey-design groups characterized by the number of preceding questionnaire modules (9–11, 12, or 13–15) to proxy respondent burden and the number of weeks of interviewer training (1–3, 4, or 5–6) to proxy interviewer skills.

### Implications for Comparative Research and Global Monitoring of Controlling Behaviors

Our findings have several implications for cross-national research and monitoring of controlling behavior as a dimension of psychological IPV against women. First, further secondary analyses may be conducted to assess whether the observed measurement non-invariance across survey-design groups is reduced with adjustment for other theoretically relevant variables, such as the survey year to adjust for potential temporal differences in item meaning; language of the questionnaire (which is available) and the primary language of the participant, interviewer, and interview (which are not available) to adjust for linguistic sources of variation in item meaning (38). Guidelines for translating scales into other languages exist (39), but suggested steps are resource intensive and may not be fully implemented in many surveys. Finally, covariates for other relevant survey conditions may include: the nature and extent of interviewer-participant matching, survey-team sizes or the extent of interviewer supervision, the expected daily quota of completed interviews per interviewer, duration of the fieldwork, and the season or conditions of the fieldwork. Many of these potential sources of non-invariance are modifiable and should be tested systematically for their contribution to measurement non-equivalence of controlling behavior items, and psychological IPV against women more generally.

Second, we recommend that global researchers with the appropriate expertise advance theory and basic research on controlling behavior, and psychological IPV against women, more broadly. The promotion of standard definitions, such as the working definition we propose, may inform refinement of existing DHS controlling behavior items in ways that improve measurement invariance of the two most non-invariant items as well as the other three items that also exhibited low invariance. Cross-cultural qualitative research may inform revisions to the wording of items in the current set that align better with how lay women conceptualize controlling behavior. Such research also may identify other cross-culturally salient controlling behaviors that reflect more fully its definition, as *one partner’s credible demands for compliance with behaviors that systematically constrain the other partner’s* actions, relationships, and activities. For example, the current set of DHS controlling-behavior items emphasize constraints on a woman’s *relationships*, and new items may operationalize constraints on a woman’s *actions* and *activities*. Survey experiments to understand how variations in survey design may causally affect the measurement invariance of the current DHS items and newly identified items also is warranted. Finally, we recommend that rigorous psychometric assessment, applying the sequenced steps we have performed here, alongside randomized experiments, become the standard for assessing the measurement invariance of all item sets that are designed to capture women’s experiences of IPV in cross-cultural research.

Despite the limitations of existing measures, current evidence suggests that controlling behaviors are among the most common forms of IPV against women globally, with substantial health implications for women (1). Thus, efforts to improve existing measures of controlling behavior, and psychological IPV against women more generally, remain critical.

## Conclusions

The Demographic and Health Surveys item set for controlling behaviors is configurally invariant across nine survey-design groups in 373,167 women who took part in 42 DHS conducted in 19 LMICs during 2005–2019. However, these items are neither scalar invariant, partially invariant, nor approximately invariant across these survey-design groups, and adjustment for woman-, population-, and national-level covariates do not account for the observed non-invariance. When administering the DVM, the DHS may consider reducing the number of prior modules to reduce respondent burden and requiring a standard number of weeks of training to ensure interviewer quality. Other invariance analyses of DHS data on controlling behaviors may adjust post-hoc for other potential sources of non-invariance, such as the ethnicity and language of respondents. Basic theoretical and empirical work still may be needed to improve items in the current set and to add items that capture other elements of our working definition for controlling behavior. Until then, the DHS controlling-behavior items are not recommended for cross-national, cross-time comparisons, including those related to monitoring SDG 5.2.1.

## Figures and Tables

**Table 1.** Characteristics of survey-design groups used for strict and approximate invariance testing of five controlling behavior items administered in 42 Demographic and Health Surveys in 19 low- and middle-income countries during 2005–2019

		# of Countries^1^	# of DHS	# of Respondents Completing any Controlling Behavior Items	# of Respondents Completing all Controlling Behavior Items	Weeks of Enumerator Training Range in Group	# of Preceding Modules Range	Attitudes about Wife Beating^2^ Mean (SD)	Years of Schooling Completed^2^ Mean (SD)	Women’s Business and Law Index Mean (SD)
Group	Group Definition	N=19	N=42	N=373,167	N=368,837	N=42	N=42	N=373,167	N=373,167	N=42
1	Highest tertile preceding modules; lowest tertile weeks of training	5	6	84,357	83,448	1–3	13–14	0.25 (0.34)	5.70 (5.04)	64.40 (8.99)
2	Highest tertile preceding modules; middle tertile weeks of training	3	3	11,368	11,282	4–4	14–15	0.35 (0.39)	4.80 (4.92)	64.60 (14.03)
3	Highest tertile preceding modules; highest tertile weeks of training	1	1	3,120	3,055	5–5	13–13	0.51 (0.36)	1.48 (3.36)	54.40 (0.00)
4	Middle tertile preceding modules; lowest tertile weeks of training	4	4	34,258	33,699	2–3	12–12	0.22 (0.33)	5.52 (4.92)	69.23 (15.48)
5	Middle tertile preceding modules; middle tertile weeks of training	4	5	48,240	47,711	4–4	12–12	0.27 (0.36)	5.96 (5.93)	66.98 (5.67)
6	Middle tertile preceding modules; highest tertile weeks of training	1	1	6,851	6,798	5–5	12–12	0.07 (0.18)	11.21 (3.91)	31.90 (0.00)
7	Lowest tertile preceding modules; lowest tertile weeks of training	5	6	99,351	98,276	2–3	9–11	0.23 (0.32)	6.36 (5.35)	69.28 (15.04)
8	Lowest tertile preceding modules; middle tertile weeks of training	9	10	38,760	38,395	4–4	10–11	0.15 (0.29)	6.59 (5.14)	55.76 (13.12)
9	Lowest tertile preceding modules; highest tertile weeks of training	5	6	46,862	46,173	5–6	9–11	0.11 (0.26)	6.99 (4.99)	72.93 (9.39)
2 & 3	[Groups combined due to small size of Group 3]	4	4	14,488	14,337	4–5	13–15	0.39 (0.39)	4.09 (4.82)	62.05 (12.54)
5 & 6	[Groups combined due to small size of Group 6]	5	6	55,091	54,509	4–5	12–12	0.25 (0.35)	6.61 (5.68)	61.13 (15.19

1 The column sum does not equal 19 because surveys from the same country conducted in different years may appear in different survey-design groups.

2 Estimates are unweighted.

**Table 2.** MGCFA invariance tests for controlling behavior items across survey design groups, without and with adjustment for individual- and national-level covariates

Model #	Woman-Level Covariates		National-Level Covariates			Survey-Design Groups (#)	Invariance Testing Model Results across Survey-Design Groups						
	Attitudes about Wife Beating^1^	Completed Years of Schooling^2^	Women’s Mean Attitudes about Wife Beating^3^	Women’s Mean Completed Years of Schooling^4^	Women’s Business, & Law Index Score^5^		Model	RMSEA	CFI	TLI	X^2^ Difference	DF	p-value
1						9	a. Configural	0.056	0.972	0.944			
							b. Scalar	0.051	0.965	0.955			
							Δ (Configural–Scalar)	**0.005**	0.007		1462.000	24	<.0001
2		X				9	a. Configural	0.038	0.976	0.962			
							b. Scalar	0.036	0.960	0.965			
							Δ (Configural–Scalar)	**0.002**	0.016		4761.464	68	<.0001
3	X					9	a. Configural	0.037	0.954	0.937			
							b. Scalar	0.039	0.925	0.932			
							Δ (Configural–Scalar)	**−0.002**	0.029		7201.342	68	<.0001
4	X	X				9	a. Configural	0.033	0.972	0.959			
							b. Scalar	0.032	0.959	0.962			
							Δ (Configural–Scalar)	**0.001**	0.013		4182.717	68	<.0001
5					X	7	a. Configural	0.05	0.958	0.932			
							b. Scalar	0.042	0.947	0.953			
							Δ (Configural–Scalar)	0.008	0.011		6009.496	54	<.0001
6			X			7	a. Configural	0.047	0.963	0.941			
							b. Scalar	0.042	0.948	0.954			
							Δ (Configural–Scalar)	**0.005**	0.015		5912.576	54	<.0001
7				X		7	a. Configural	0.048	0.963	0.940			
							b. Scalar	0.040	0.951	0.957			
							Δ (Configural–Scalar)	0.008	0.012		5580.935	54	<.0001
8			X	X	X	7	a. Configural	0.039	0.946	0.924			
							b. Scalar	0.037	0.929	0.931			
							Δ (Configural–Scalar)	**0.002**	0.017		866.012	54	<.0001
9	X	X	X	X	X	7	a. Configural	0.034	0.939	0.919			
							b. Scalar	0.034	0.931	0.919			
							Δ (Configural–Scalar)	**0.000**	0.008		1599.197	24	<.0001

1 Scored as a mean of five dichotomous items, each indicating whether the respondent finds wife beating acceptable in that situation

2 Total years of schooling completed

3 Average of individual-level scores for all respondents from each country

4 Average of total years of schooling completed for all respondents from each country

5 The Women, Business, and Law Index scores 35 “data points . . . across eight indicators of for our five binary questions, with each indicator representing a different phase of a woman’s career”.

The overall WBLI represents unweighted averages of each indicator, scaled to 100, with 100 representing the highest possible score.

**Table 3.** Thresholds, loadings, and R2 values from alignment optimization analysis of controlling behaviors items using the full pooled sample of Demographic and Health Surveys split into nine groups based on survey design

	Thresholds		Loadings	
Controlling-behavior Items	Weighted average value across invariant groups	R^2^	Weighted average value across invariant groups	R^2^
Is jealous or angry if she talks to other men?	−2.24	0.00	1.99	0.31
Frequently accuses her of being unfaithful?	0.10	0.31	2.64	0.27
Does not permit her to meet her female friends?	0.48	0.00	2.74	0.00
Tries to limit her contact with her family?	1.31	0.00	2.75	0.00
Insists on knowing where she is at all times?	−1.10	0.00	2.05	0.57
Non-invariant parameters out of total	23 of 45 (51%)		12 of 45 (27%)	
Total non-invariant parameters	35 of 90 (38.9%)			

## Data Availability

The data that support the findings from this study are available for download upon completing the new user registration and data access request form from the Demographic and Health Surveys at the following website: https://www.dhsprogram.com/data/new-user-registration.cfm

## Abbreviations

AO: Alignment optimization
CFI: Comparative fit index
DHS: Demographic and Health Survey
DVM: Domestic violence module
IPV: Intimate partner violence
LMIC: Low- and middle-income country
MGCFA: Multiple group confirmatory factor analysis
PSU: Primary sampling unit
RMSEA: Root mean square error of approximation
SD: Standard deviation
SDG: Sustainable Development Goal
USAID: United States Agency for International Development
TLI: Tucker Lewis Index
WBLI: Women, Business, and Law Index
WLS: Weighted least squares
